# Biological Network Approaches and Applications in Rare Disease Studies

**DOI:** 10.3390/genes10100797

**Published:** 2019-10-12

**Authors:** Peng Zhang, Yuval Itan

**Affiliations:** 1 St. Giles Laboratory of Human Genetics of Infectious Diseases, Rockefeller Branch, The Rockefeller University, New York, NY 10065, USA; 2 The Charles Bronfman Institute for Personalized Medicine, Icahn School of Medicine at Mount Sinai, New York, NY 10029, USA; yuval.itan@mssm.edu; 3 Department of Genetics and Genomic Sciences, Icahn School of Medicine at Mount Sinai, New York, NY 10029, USA

**Keywords:** biological network, bioinformatics, database, software, application, rare diseases

## Abstract

Network biology has the capability to integrate, represent, interpret, and model complex biological systems by collectively accommodating biological omics data, biological interactions and associations, graph theory, statistical measures, and visualizations. Biological networks have recently been shown to be very useful for studies that decipher biological mechanisms and disease etiologies and for studies that predict therapeutic responses, at both the molecular and system levels. In this review, we briefly summarize the general framework of biological network studies, including data resources, network construction methods, statistical measures, network topological properties, and visualization tools. We also introduce several recent biological network applications and methods for the studies of rare diseases.

## 1. Introduction

Network biology provides insights into complex biological systems and can reveal informative patterns within these systems through the integration of biological omics data (e.g., genome, transcriptome, proteome, and metabolome) and biological interactome data (e.g., protein-protein interactions and gene-gene associations). Collectively, through the applications of statistics, graph theory methods, mathematical modeling, and visualization tools, network biology has deepened our understanding of biological mechanisms [1,2] and diseases etiologies [3,4], and has facilitated therapeutics discovery [5,6].

**Figure 1:**
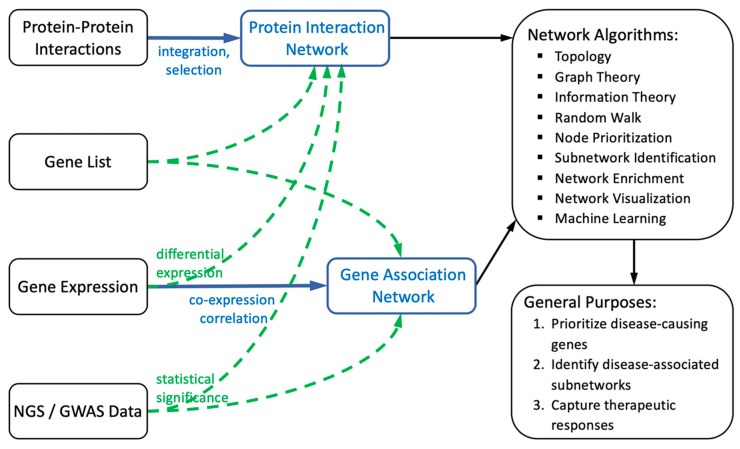
A flowchart illustrating the general framework of biological network studies. The blue arrows and text correspond to the construction of biological networks, whereas the green arrows and text correspond to the mapping of biological data onto the network.

Biological networks can be constructed and applied in a number of ways to address biological questions. Some of the general purposes of biological networks include: (1) identification and prioritization of disease-causing candidate genes [7,8]; (2) identification of disease-associated subnetworks and systematic perturbations of diseases [3,9]; and (3) capturing therapeutic responses to facilitate target identification and drug discovery [5,6]. In this review article, we will briefly introduce the general framework of biological network approaches, followed by several associated applications and methods for studying rare diseases. Figure 1 illustrates a flowchart of biological network studies.

## 2. General Framework of Biological Networks

### 2.1. Construction of Biological Networks

#### 2.1.1. Protein Interaction-Based Network Construction

Protein-protein interactions (PPIs) are widely used in the construction of biological networks. The source of PPIs can be obtained from large-scale experiments, computational predictions, and the mining of published literature. Physically interacting PPIs have often been used to generate the global reference biological network, and have been used as a starting point for further analyses. Several widely used databases (e.g., BioGRID [10]), IntAct [11], STRING [12], HIPPE [13], and HPRD [14]) provide human physical PPIs (Table 1), and one of them, STRING, also provides gene associations and interactions predicted by text-mining, with pre-computed confidence scores. BioGRID, IntAct, and STRING are the resources updated most frequently. The simplest biological entities can be mapped onto PPI networks, including lists of genes of interest, known or candidate disease-causing genes, approved or investigational therapeutic targets, and diagnostic biomarkers.

Tissue-specific PPI networks have been shown to provide significantly improved biological enrichment compared to the global network [15,16], particularly for the prioritization of disease genes and for the study of disease progression [7,17,18]. Tissue-specific PPI networks can be constructed by mapping the gene expression data obtained in transcriptome or proteome experiments onto the global PPI network, or they can be obtained from databases providing tissue-specific interactions/networks (e.g., TissueNet [19], GIANT [20], IID [21], and TISPIN [22]). These resources (Table 1) offer various approaches and features for studying tissue-specific systems and activities. When studying diseases affecting multiple tissues (e.g., autoimmune disorders), additional biological data are required for the derivation of multiple tissue-specific networks and for the comparison of disease-associated signals between tissues. Network-based therapeutics investigations have recently moved into the limelight. These studies typically integrate tissue-specific protein interaction networks and differential gene expression data to represent the drug perturbation signatures in different molecular environments, and this approach has greatly improved drug target identification and drug response prediction [20,23,24,25].

#### 2.1.2. Gene Correlation/Association-Based Network Construction

Genes can also be connected to each other on the basis of the correlation in their levels of expression, or the statistical significance of association with a given phenotype, to construct the gene-gene networks. Such networks are usually context-specific (disease-specific, tissue-specific, cell type-specific, treatment-specific, etc.) based on the condition in which the experiments were designed and performed. Gene-gene correlations/associations can be inferred by various methods, including gene co-expression correlation [26], Bayesian probability [27], Gaussian graphical model [28], or information theory method [29,30]. The inferred biological network may be binary if gene pairs are retained above a given cutoff value. However, the choice of cutoff can have a major effect on the structure of the resulting network, thus possibly affecting the downstream network analysis. The biological network can also be edge-weighted by assigning the correlation/association value to each gene pair, which increases the complexity of the network and the computing time required. Such gene-gene networks can be constructed from in-house experimental data, or can be based on transcriptome or proteome data provided by public databases (e.g., TCGA [31], GTEx [32], SRA [33], GEO [34], ArrayExpress [35], and HumanProteinAtlas [36]). 

### 2.2. Computations of Biological Networks

#### 2.2.1. Topological Properties

Given a network structure, the established graph theory provides a number of topological properties for the local description of all nodes and the global characterization of the entire network or a subnetwork. These topological properties were initially developed for studies in the fields of sociology, physics, mathematics, and computer sciences to characterize the connectivity, organization, efficiency, and stability of complex networks. For instance, the centrality properties represent the degree of influence of a person in sociological studies [37], the clustering coefficient reveals the structural relations in interpersonal relationships [38], and the PageRank algorithm is used in Google searches to sort the results according to the webpage connections and Internet popularity [39]. Over the last decade, many established topological properties have been successfully employed in biological network studies [2,40]. Table 2 summarizes several typical local and global topological properties that have been used to reveal biological implications.

Several tools for calculating the network-based local and global topological properties are current available, including the PROFEAT webserver [54], Python library NetworkX [55], R packages igraph [56], and QuACN [57]. The documentation on PROFEAT currently provides the most diverse and comprehensive collection of algorithms for these properties [54].

#### 2.2.2. Node Prioritization

One popular use of biological networks is node prioritization (e.g., genes) in the network, particularly for the identification of disease-causing genes or therapeutic targets in a context-specific network. The simplest method is to rank all genes by a certain local topological property (e.g., degree, closeness centrality, and PageRank centrality). However, biological systems are often very complex, and the use of one or a few properties is, therefore, less desirable for this purpose.

Expression-based methods generally start with the mapping of gene expression data to a global reference network or a context-specific network to generate expression weighted networks. For example, the PINTA method accepts disease-specific gene differential expression values as input, and then searches for candidate genes that tend to be surrounded by many differentially expressed genes in a genome-wide PPI network derived from the STRING database [58]. PINTA implements five random walk algorithms (heat kernel ranking, diffusion ranking, random walk, HITS, and k-step Markov), which consider both the strength of the interactions and the differential expression levels of the interacting genes, for searching and prioritizing the disease-specific candidate genes [58].

The whole genome/exome sequencing or directed panel sequencing data from patients can be used for network analysis as well. The genomic variants can be filtered by population genetics (e.g., gnomAD [59] and 1,000 Genomes Project [60]) and damage predictions (e.g., CADD [61] and PolyPhen-2 [62]) to obtain a shortlist of variants having minor allele frequencies compatible with the disease prevalence and a relatively high damaging prediction. PopViz [63] is an online tool that unifies gnomAD and CADD, which can be helpful in the step of variant filtration and gene selection. The genes harboring these likely damaging variants could be mapped onto the PPI-based network to check their relationships with the known disease-causing genes, and to search for gene clusters encompassing these genes followed by functional pathway and gene ontology enrichment.

In the case of using genome-wide association studies (GWAS) data, association-based methods often start with the testing of statistical associations between the single-nucleotide polymorphisms (SNPs) and phenotypes. The SNPs with genome-wide statistical significance are considered to be phenotype-associated, and are then are mapped to a global reference network or a context-specific network. Most studies first identify the known disease-causing genes in the GWAS-inferred networks, and then apply topological similarity [64], information flow [64], random walk [8], or network propagation [65] to identify and prioritize new candidate genes.

#### 2.2.3. Subnetwork Identification

Networks are often naturally split into modules or subnetworks [66]. Subnetwork identification usually begins with a search with one or more seed genes, and then expands to the neighboring nodes. This expansion continues with the calculation of a parameter controlling the gene clusters until a threshold is met. The detection of such subnetworks in disease conditions or drug treatments can provide valuable insight into disease etiology or therapeutic responses.

An example of expression-based subnetwork analysis is provided by a recent study that overlaid islet-specific gene expression data from microarray and RNA-seq onto a protein interaction network to investigate the dysregulation of type-II diabetes [67]. This study used a graph decomposition algorithm [68] to identify the tightly connected gene clusters by setting a minimum network density as the controlling parameter. Functional enrichment testing was also performed, and several gene subnetworks significantly associated with diabetic phenotypes were identified [67]. Another study demonstrated that the integration of interactome data with blood leukocyte gene expression data in the conditions of receiving inflammatory stimulus (endotoxin) at different time points has facilitated the identification of functional modules perturbed by exposure to endotoxin in blood leukocytes [69]. The identified gene subnetworks are responsible for innate immune system tolerance and the increase in susceptibility to infection [69].

Several association-based approaches have been developed for the identification of subnetworks significantly associated with certain phenotypes. dmGWAS is one of these approaches, which integrates the gene-based *p*-value from GWAS and the human PPI network, and then searches for candidate subnetworks with dense phenotype association signals [9]. In a study on schizophrenia, the association *p*-values were overlaid onto a global PPI network to generate a node-weighted PPI network, and the dmGWAS was applied to search for subnetworks enriched in schizophrenia [70]. Each gene was scanned by the iterative recruitment of its neighboring genes with the highest association score, and the module continued to expand until no more neighboring nodes satisfied the criteria. The result suggested SNPs in nine genes in the subnetwork were significantly associated with schizophrenia. Although this method is named as dmGWAS, it also works with differential gene co-expression data and genes harboring deleterious variants data for subnetwork identification.

### 2.3. Additional Resources to Assist the Biological Network-Based Studies

Many additional resources can also be used to assist and enrich the biological network-based studies to help to better understand and interpret the biological networks. Reactome [71] and KEGG [72] databases provide signaling pathways and their belonging genes, thus, enable us to test whether disease-associated subnetworks are significantly enriched for a particular functional pathway, which may imply the relationship between the pathway and the disease. Pathways are subsets of biological networks with specific biological functions and signaling cascades, which can also be used as small directed networks with tens of heterogeneous interacting components for network-based analysis. GO [73] defines a list of terms used to represent the properties of each gene, which can be used to extract a network of genes belonging to one or more particular biological process or molecular functions. GO can also be used to test if a disease-associated subnetwork is significantly enriched for a specific property. The ENCODE [74] and FANTOM [75] databases provide information on molecular regulators governing the gene expression, including transcriptional promoters, enhancers, repressors, and associated genes. This information can be used to define the transcriptional regulatory dependency and to integrate with the PPI or gene co-expression network for detecting the abnormal or missing regulatory signals in diseases. The HPO [76], OMIM [77], and Orphanet [78] databases provide comprehensive information on genes and genomic variants that cause diseases, therefore enabling probing and analyzing these known disease-causing genes in the network, and thus searching for new disease-causing candidate genes. They can also be used to study the disease-disease relationships based on the shared disease-causing genes and their topological properties in the network. Genepanel.iobio presents an integrated platform of Genetic Testing Registry [79] and Phenolyzer [80] to be used to generate a list of scored genes associated with one or more diseases. The TTD [81] and DrugBank [82] databases provide a collection of approved, clinical, investigational and terminated drugs, and therapeutic targets, which can help us to understand the successful and the failed drugs and therapeutic targets in a drug-target network perspective, and can help to guide the development of new drug targets for therapeutic purposes or the investigation of new uses of existing therapeutics (drug repositioning).

Biological network-based studies can also be carried out on model organisms to assist the translational studies of human diseases [83,84]. Using mice as an example, researchers can obtain mouse protein-protein interactions from resources such as the BioGRID10 and STRING12 databases, mouse gene expression data from the GXD database [85], human-mouse disease phenotype connections from the GXD database [86], and signaling pathways of mouse from the Reactome database [71]. Okada et al. combined risk genetic variants, PPI networks, functional pathways, and mouse phenotypes data to study the genetics of rheumatoid arthritis for its drug discovery [83]. Lin et al. integrated genomic variants filtering, mouse/human phenotype association scoring, and network-based scoring for risk gene prioritization in sequencing-based studies of human diseases [84].

There are several tools available to assist in network visualization, including Cytoscape [87] (the most widely used), as well as Gephi [88], NAViGaTOR [89], PINA [90], and GraphWeb [91]. These tools have various options, allowing users to import the customized network structures, choose the different network layouts, change the node/edge size and color, and highlight the subnetworks. The simplest and the most popular network file format compatible with these visualization tools is SIF (simple interaction format). In this format, each line specifies a source node, a relationship, and a target node in a tab-delimited flat text file. Other acceptable network file formats include JSON (Cytoscape.js format), NET (Pajek NET format), NNF (nested network format), GML (graph modeling language), and SBML (systems biology markup language). The network layout can be a grid, circular, hierarchical, degree-sorted, or force-based. The force-based algorithm assigns forces to the nodes, and stimulates the forces and motions between the nodes to minimize energy. The force-based layout is often preferred to optimize the graph readability, particularly for very large networks.

## 3. Applications of Biological Networks in Studying Rare Diseases

Biological networks have been applied to a broad spectrum of disease types, and we will discuss a few such studies of rare diseases. Diseases are generally considered to be rare when they affect fewer than one person in 2000, according to Genetic and Rare Diseases Information Center, U.S. National Institutes of Health (NIH) [92]. There are about 6000 rare (or orphan) diseases cataloged by the Orphanet database [78]. Rare diseases present a wide range of disease phenomena, from well-characterized monogenic origins to complex heterogeneous genetic associations. Due to the small size of patient population, high research cost, and a probable low return in revenue, the studies on rare diseases have been largely neglected. So far, the US Food and Drug Administration (FDA) has approved 492 orphan drugs for only 5% of rare diseases [93]. With the help of computational approaches, the investigations of rare diseases can be greatly accelerated for the discovery of their disease etiology and for their new therapeutics. The following sections will summarize two network-based studies on rare diseases (congenital hyperinsulinism [94] and systemic sclerosis [95]), and three network-based methods (HGC [96], Vertex-similarity [97], and DIGNiFI [98]) for searching for disease-causing genes in rare diseases (Table 3).

### 3.1. Congenital Hyperinsulinism

Congenital hyperinsulinism (CH) is a rare disease causing individuals to have abnormally high levels of insulin, affecting approximately 1 in 50,000 newborns [99]. Patients with this condition usually have serious complications such as breathing difficulties, intellectual disability, brain damage, seizures, and coma. To date, mutations in 14 genes (*ABCC8, CACNA1D, FOXA2, GCK, GLUD1, HADH, HK1, HNF1A, HNF4A, KCNJ11, PGM1, PMM2, SLC16A1,* and *UCP2*) have been found to be associated with congenital hyperinsulinism [94,100]. Mutations of the ABCC8 gene are the most commonly known cause of the disease, accounting for 40% of the cases, whereas the cause remains unknown in about half of all CH patients.

At the time of the study by Stevens et al., there were nine genes (*ABCC8*, *GCK*, *GLUD*, *HADH*, *HNF1A*, *HNF4A*, *KCNJ11*, *SLC16A1*, and *UCP2*) known as CH disease-causing that encompassed different molecular functions including transcription factors, metabolic enzymes, and solute transporters [94]. These nine genes were mapped onto the BioGRID PPI interactome [10], and they applied the modularity method (a graph-partition algorithm) [66] to these nine seed genes in the global PPI network. Surprisingly, it was found that these nine functionally diverse genes were topologically close, being clustered together in a core subnetwork enriched in cellular signaling, nuclear signaling, growth signaling, and developmental pathways. The authors suggested that the genes closely connected to these nine known genes in this identified subnetwork were potential new candidate genes worthy of study to determine the unknown etiology of congenital hyperinsulinism.

### 3.2. Systemic Sclerosis

Systemic sclerosis (SSc) is a multi-organ autoimmune disorder characterized by skin fibrosis and vascular obliteration. Its prevalence is approximately 1 in 6,500 adults, with women predominantly affected (female/male ratio of 4:1) [78]. Its precise etiology is still unknown. One recent study used multiple-network approaches to decipher the tissue-specific molecular signatures of systemic sclerosis [95]. Taroni et al. used the 573 microarray gene expression data for four tissues affected by SSc (skin, lung, esophagus, and peripheral blood) from 321 SSc patients to infer weighted gene co-expression networks by using the WGCNA R package [101]. They first identified the disease-associated subnetworks overlapping across tissues by a consensus clustering procedure [102], and then found a common pathogenic signature related to the immune-fibrotic axis in multiple tissues, suggesting that pro-fibrotic macrophages are present in the tissues of SSc patients. Then the GIANT database was queried to retrieve the tissue-specific networks by using the immune-fibrotic axis gene sets, and the subnetworks were detected by the igraph R package [56]. To understand the difference of the immune-fibrotic connectivity between lung and skin, differential network analysis was performed by contrasting the detected lung and skin subnetworks, and distinct transcriptional programs were identified for macrophages activation in the affected lungs from SSc patients.

### 3.3. HGC and Its Application to Herpes Simplex Virus Encephalitis

Herpes simplex virus encephalitis (HSE) is a rare disorder in which the central nervous system is infected with the Herpes simplex virus (HSV). According to the Orphanet database, HSE has an annual incidence of 1/250,000–1/500,000. It frequently involves the frontal and temporal lobes, leading to personality changes, cognitive impairment, aphasia, seizures, and focal weakness [78]. Genetic studies on HES patients have revealed that *TLR3*-deficiency underlies HSE pathogenesis in a fraction of children affected with this disease [103].

Motivated by searching for more HSE-causing candidate genes, Itan et al. proposed a network-based method, the Human Gene Connectome (HGC), for identifying closely related gene clusters centered on a given gene of interest [96]. HGC extracted the direct human PPIs (166,468 connections for 12,009 genes, extended to 328,391 connections for 14,129 genes in the updated version) from the STRING database [12], and calculated gene-to-gene distances by inverting the gene-to-gene association scores provided by the database. The shortest path distances and the corresponding routes were calculated for all possible pairs of human genes with NetworkX [55]. Finally, HGC constructed a gene-centric connectome for all human genes, taking the shortest path distances, distance distribution, and *p*-value for the proximity of a peripheral gene to the central gene into account. In the *TLR3*-centric connectome, 20 of the 21 known HSE-causing genes are identified as being among the top 5% of the closet neighbors of *TLR3*, according to its latest version. These genes include *TRIF*, *TICAM2*, *UNC93B1*, *TRAF3*, *TBK1*, and so on. These findings are supported by extensive studies of the etiology of HSE [103,104,105,106]. HGC has recently been shown to predict novel primary immunodeficiency (PID) candidate genes based on functional relatedness to all known PID genes [107].

### 3.4. Vertex-Similarity and Its Application to 172 Rare Diseases

This method first proposes an algorithm to compute the vertex-similarity (VS) score between each pair of vertices (e.g., genes) in a given network [97]. When two genes are directly connected, their similarity score is calculated with an edge-weighted equation, taking the neighborhood into account. When two genes are not directly connected, their similarity score is calculated by a shortest-path-based equation. Therefore, for a given a disease with known causal genes as seed genes, the VS algorithm ranks all other genes according to the computed VS scores with the seed genes.

The authors constructed a human protein interaction network containing 11,765 proteins and 69,167 interactions by compiling three databases (HPRD [14], BIND [108], and Reactome [71]) and data from three relevant publications [49,109,110]. They tested the VS method on 1598 known orphan disease-causing genes for 172 orphan diseases, with data obtained from the Orphanet database [78]. They performed leave-one-out cross validation by selecting a causal gene for one rare disease as the target gene and mixing it with 99 randomly selected genes to form a test set of 100 genes. The remaining disease-causing genes for the disease concerned were used as the training set, and the 100 test genes were then ranked by the VS method and two other gene prioritization methods (PageRank and Interconnectedness). The success rate was then evaluated by examining whether the target gene was ranked in the top-*k* genes of the test set of 100 genes. This validation ran iteratively by assigning each disease-causing gene as the target gene for all 172 rare diseases. The *k* parameter was screened from 1 to 20, and the VS method had a success rate ranging from 43% to 68%, revealing a better performance than the other methods [97].

### 3.5. DIGNiFI and Its Application to 128 Rare Diseases

The DIGNiFI (Disease Causing Gene Finder) method computes the topological similarity between genes based on local and global connected paths in the PPI network [98]. Like the VS method, it calculates the gene-gene similarity in two ways (one for directly connected genes, and the other for indirectly connected genes) to generate a direct neighbor (DN) score that reflects the local connectivity. It also uses the local random walk (LRW) algorithm, a modified random walk method for large and sparse networks, to identify the global network features. DIGNiFI ranks the candidate genes of a given disease by combining the DN and LRW scores.

The authors constructed a human PPI network with 9,453 proteins and 36,867 interactions from the HPRD database [14] and used 128 orphan diseases with 1184 disease-causing genes from the Orphanet database [78] to test their method. With the same validation approach as for the VS method, DIGNiFI outperformed the VS method and several other methods for the prioritization of disease-causing genes for rare diseases. Moreover, gene prioritization can be improved further by the use of gene ontology annotations and protein complex information to refine the PPI network. Its success rate can reach 50% to 75% for scanning the top-*k* parameter from 1 to 10.

## 4. Discussion and Conclusions

Recent studies have demonstrated the broad utility of the network concept for addressing questions in systems biology, disease etiology, and therapeutics discovery. Although this review focused on several applications for rare diseases, these approaches could mostly be tailored for the study of complex/common diseases, and there are many studies and reviews of biological network approaches that address complex diseases [111,112,113]. With a solid theoretical foundations and successful biological applications, network biology is becoming emergingly popular. Progress towards the broader and better use of biological networks will require the integration of more layers of biological omics data, the recruitment of tissue-/cell type-specific information, the characterization of more graph-theory properties, and network enrichment with the knowledge of signaling pathways and gene ontology. The development of appropriate integrations of biological networks and machine learning or deep learning algorithms for systematic modeling and prediction purposes is anticipated.

The study of biological networks still poses several major challenges. Most studies are based on protein networks, but PPI networks are static, lacking spatial and temporal information about the interactions, and are limited by the coverage and quality of the interactions. The PPIs available to date are far from complete, and they contain many false positive interactions. Moreover, human diseases (common or rare, complex or simple) can be caused by a single strong signal, or by a number of weaker signals acting together. The improvement of current methods or the creation of new methods for identifying the disease-causing signals hidden within multiple connections with higher sensitivity and specificity remains a challenge, as well as an opportunity.

As demonstrated in this article, network biology studies are highly diverse, differing in terms of data types, data collection and preprocessing, statistical tests, mathematical models, and the purposes of the various specific studies. This review aims to provide a general framework, and highlights the key concepts and components of biological network studies. The approaches and the applications mentioned in this review were usually developed for particular types of biological data and phenotypes, but those methodologies can often be adapted and reformulated for other types of data and phenotypes with appropriate design.

## Figures and Tables

**Table 1:** Databases providing human protein-protein interaction data.

**Global PPI databases**				
**Database**	**Type of Interaction**		**Number of Interactions**	**Number of Genes/Proteins**
BioGRID [10]	Physical		371,513	23,795
IntAct [11]	Physical		379,393	25,643
STRING [12]	Physical, association, text-mining		11,759,455	19,567
HIPPE [13]	Physical		273,900	17,000
HPRD [14]	Physical		41,327	30,047
**Tissue/cell-type-specific PPI databases**				
**Database**	**Type of Interaction**	**Number of Tissues/Cell-Types**	**Number of Interactions**	**Number of Genes/Proteins**
TissueNet [19]	Physical	40	243,706	17,283
GIANT [20]	Physical, co-expression	144	n.a.	n.a.
IID [21]	Physical, predicted	26	975,877	19,250
TISPIN [22]	Physical	53	128,579	13,123

**Table 2:** Typical local and global topological properties (with their computational equations and graph theory explanation) that have been used in biological studies.

Level	Topological Property	Computational Equation	Graph Theory Explanation	Biological Implication
Local	Clustering coefficient [41]	$2e_{i}/\deg_{i}\left(\deg_{i}-1\right)$, deg_i_ is the number of interacting partners of a node, and e_i_ is the number of links among all neighbors of a given node.	Measures the tendency of a node to form a group with the neighboring nodes.	Used to analyze the organizational properties of human protein networks [42], and to validate the association of a drug with existing proteins in the drug-target network [6].
Local	Closeness centrality [43]	$1/\left(\overset{N}{\underset{j=1}{\sum }}D_{\mathrm{ij}}/N\right)$, D_ij_ is the shortest path length from node i to j, and N is the number of nodes in the network.	Measures how fast information can spread from a given gene to the other reachable genes.	These centralities have been used to prioritize disease candidate genes [44], identify important genes in drug discovery [45], and shed light on disease-disease relationships [46].
Local	Betweenness centrality [47]	$\underset{s\neq i\neq t}{\sum }\sigma_{\mathrm{st}}\left(i\right)/\sigma_{\mathrm{st}}$, σ_st_(i) is the number of shortest paths from s to t passing through gene i, and σ_st_ is the number of shortest paths from s to t.	Indicates the number of times a given node serves as a linking bridge on the shortest path between any other two nodes.	
Local	PageRank centrality [39]	$1-d/N+d\overset{N}{\underset{j=1}{\sum }}(A_{\mathrm{ij}}\cdot {\mathrm{pRank}}_{j}/\deg_{j})$ Initializes each node’s centrality to an equal probability value 1/N, then iteratively updates each node’s centrality by a damping factor d, the number of neighbors, and the neighbors’ centrality. It stops when PageRank centrality converges.	Gauges the importance of a given node by considering both the number of connections of the nodes, and the importance of the connected nodes.	PageRank centrality has been used to identify protein targets in metabolic networks [48], and candidate marker genes for prognosis prediction in patients with pancreatic cancer [49].
Global	Connectivity centralization [50]	$\left(N/\left(N-2\right)\right)\cdot \left(\max\left(\deg_{i}\right)/(N-1\right)-\;2E/\left(N\left(N-1\right)\right))$, E is the number of edges in the network.	Distinguishes highly connected networks or decentralized networks.	Used in studies of the structural differences between metabolic networks [51].
Global	Heterogeneity [50]	$\surd \left(\left(N\overset{N}{\underset{i=1}{\sum }}\left(\deg_{i}{}^{2}\right)/{\left(\overset{N}{\underset{i=1}{\sum }}\deg_{i}\right)}^{2}\right)-1\right)$.	Measures the variation of the connectivity distribution.	Reflects the tendency of a network to have hub genes [50].
Global	Global efficiency [52]	$\left(\overset{N}{\underset{i\neq j}{\sum }}1/D_{\mathrm{ij}}\right)/\left(N\left(N-1\right)\right).$	Represents the information exchange efficiency across the entire network or a defined subnetwork.	Used to describe the brain neuro-connectivity [53].

**Table 3:** The applications and methods of biological networks in studying rare diseases.

Application/Method	Source of Network	Algorithm	Results
Congenital hyperinsulinism [94]	PPI network (BioGRID)	Graph-partitioning for subnetwork identification.	Identified the nine known disease-causing genes that are functionally diverse and clustered together in a core subnetwork.
Systemic sclerosis [95]	Gene co-expression network	Consensus clustering and differential network analysis for subnetwork identification.	Identified common pathogenic signature in four tissues of systemic sclerosis patients, and identified a distinct disease process in the lung.
HGC [96]	Gene association network (STRING)	Shortest path distance, distance distribution, and statistical significance.	Identified 20 of the 21 known disease-causing genes of herpes simplex virus encephalitis, and further used to identify the disease-causing genes of primary immunodeficiency diseases, which were experimentally validated.
Vertex Similarity [97]	PPI network (3 papers, HPRD, BIND, Reactome)	Pairwise similarity by an edge-weighted and neighbor-considered equation for connected nodes, or a shortest-path-based equation for disconnected nodes.	Developed the Vertex Similarity method to identify and rank orphan disease candidate genes of 172 rare diseases based on the known disease-causing genes in the protein interaction network.
DIGNiFI [98]	PPI network (HPRD)	Pairwise similarity by measuring local direct neighbor connectivity, and global network feature by a random walk algorithm.	Developed DIGNiFI method to discover causative genes in orphan diseases of 128 rare diseases, and suggested the use of GO terms and protein domains to refine PPI networks.
